# The Impact of Cyanobacteria Blooms on the Aquatic Environment and Human Health

**DOI:** 10.3390/toxins14100658

**Published:** 2022-09-23

**Authors:** Weizhen Zhang, Jing Liu, Yunxing Xiao, Yumiao Zhang, Yangjinzhi Yu, Zheng Zheng, Yafeng Liu, Qi Li

**Affiliations:** 1 School of Ecological Environment, Chengdu University of Technology, Chengdu 610059, China; 2 Department of Environmental Science and Engineering, Fudan University, Shanghai 200433, China

**Keywords:** cyanobacteria blooms, cyanobacteria toxins, aquatic environment, human health

## Abstract

Cyanobacteria blooms are a global aquatic environment problem. In recent years, due to global warming and water eutrophication, the surface cyanobacteria accumulate in a certain area to form cyanobacteria blooms driven by wind. Cyanobacteria blooms change the physical and chemical properties of water and cause pollution. Moreover, cyanobacteria release organic matter, N (nitrogen) and P (phosphorus) into the water during their apoptosis, accelerating the eutrophication of the water, threatening aquatic flora and fauna, and affecting the community structure and abundance of microorganisms in the water. Simultaneously, toxins and carcinogens released from cyanobacteria can be enriched through the food chain/web, endangering human health. This study summarized and analyzed the research of the influence of cyanobacteria blooms on the aquatic environment and human health, which is helpful to understand further the harm of cyanobacteria blooms and provide some reference for a related research of cyanobacteria blooms.

## 1. Introduction

Cyanobacteria are widely distributed in marine and freshwater, and have more robust adaptability than most eukaryotes [1]. They have the ability to grow and reproduce in extreme environments (ice and snow, hot springs, alkaline soda lakes, brine pools, deserts, and polar regions) [2]. When organic matter, N (Nitrogen), P (Phosphorus), and other nutrients are enriched in water, cyanobacteria multiply and accumulate into dominant groups. The cyanobacteria blooms, which form green, red-brown, and red in freshwater or marine, are one of the most notable symptoms of nutrient enrichment or eutrophication [3]. Cyanobacteria blooms are becoming increasingly common worldwide and pose a serious threat to the sustainability of aquatic ecosystems, such as Taihu Lake in China, Lake Erie in the United States, Lake Winnipeg in Canada, and Lake Nieuwe Meer in the Netherlands [4]. Since the 1930s, plenty of studies have been carried out on cyanobacteria blooms, including the causes of cyanobacteria blooms [5], the harm of cyanobacteria products and the symbiosis of algae and bacteria [6], and the nutrient effect of cyanobacteria blooms [7].

Cyanobacteria blooms will cause hypoxia in the water, as they accumulate and decompose, resulting in various toxic secondary metabolites and other harmful compounds (such as toxins, hydrogen sulfide, and odor substances) [8], which have an impact on the aquatic flora and fauna, and the community structure and quantity of microorganisms [9]. Cyanobacteria blooms not only damage aquatic creatures but also endanger human health. Direct or indirect contact with cyanobacterial toxins lead to acute gastroenteritis, respiratory adverse reaction, skin rash, oral ulcer, and other diseases [10], and even induce cancer [11].

## 2. The Pollution of Cyanobacteria Blooms to Water

It is considered that cyanobacteria blooms form when the cyanobacteria reaches 10^5^ cells/mL, or the chlorophyll a (Chla) concentration reaches 10 μg/L, and a visible covering layer forms on the surface of the water [12]. The cyanobacteria blooms’ decay process has a more serious impact on the aquatic environment. Aerobic and anaerobic reactions exist in the degradation process of cyanobacteria, and toxins and odorous gases are released. During the decomposition of cyanobacteria blooms, a large number of organic substances and soluble nutrients will be released to water, which will lower the transparency of water, aggravate the eutrophication of water, and form “black spots” [13]. Cyanobacteria blooms will lead to the acidity of the water, the rising trend of conductivity, the continuous increase in chemical oxygen demand, and the increase in organic matter concentration in the water [14]. In addition, organic debris formed by cyanobacteria accumulation has a high decomposition rate in the water, which can be decomposed by 41.9% within 48 h [15], which will harm the ecosystem of the water [12,16]. A large amount of dissolved organic matter (DOM) is released during the decline of cyanobacteria, and with the progress of the reaction, dissolved organic carbon (DOC) is converted into dissolved inorganic carbon (DIC), and most of them are, lastly, transformed into humus, which is challenging to degrade [17].

**Figure 1:**
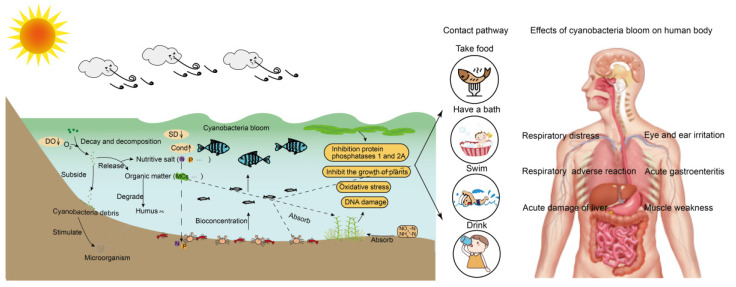
The main hazards of cyanobacteria bloom to water bodies, aquatic organisms, and the human body. (DO: dissolved oxygen; SD: water transparency; Cond: conductivity; N: nitrogen; P: phosphorus; MCs: microcystins).

Cyanobacteria can accelerate the migration and transformation of different forms of P in water during a recession [18]. Through sorting out and analyzing the monitoring data of P concentration in Taihu Lake from 1949 to 2020, it was found that the P concentration in Taihu Lake had significant natural fluctuation [19]. It was found that the concentration of P in lakes has a negative impact on water transparency (SD) (Figure 1) [20]. In addition, cyanobacteria blooms can play an important role in the movement of nutrients in aquatic ecosystems (Figure 1) [21].

### 2.1. Impacts of Cyanobacteria Blooms on Aquatic Fauna

During cyanobacteria blooms, a large number of dead cyanobacteria will sink to the bottom and decompose, consuming oxygen, which will reduce the dissolved oxygen (DO) in water, thus affecting the living conditions of aquatic fauna, causing the disappearance of some fish, shellfish, and invertebrates, and decreasing the species diversity of the aquatic ecosystem [22]. In the apoptosis of cyanobacteria, secondary metabolites such as toxins, odorous substances, and other substances were released into the water, and the concentration of ammonia (NH_4_^+^) and microcystins (MCs) will increase simultaneously, causing acute or chronic adverse effects on aquatic organisms [23].

Exposure to MCs leads to lipid peroxidation, DNA damage, and changes in antioxidant enzymes, such as superoxide dismutase (SOD), peroxidase (POD), and catalase (CAT) in different aquatic organisms. MCs can cause damage to their circulatory system, digestive system, and immune system. Simultaneously, they will induce changes in detoxification enzymes such as glutathione S-transferase (GST) and glutathione peroxidase (GPx) [24]. Studies have demonstrated that the liver is the main target of MCs (Figure 1) [25]. By studying the bioaccumulation law of MCs in two snails in a cyanobacteria-bloom plateau lake, it was found that the hepatopancreas was the main target of two snails (Figure 1) [26]. Some reports indicate that the concentration of MCs in the intestines, gonads, and muscles of *Cyprinus carpio* was lower but higher in hepatopancreas [27]. MCs are enriched in the hepatopancreas of *Macrobrachium rosenbergii*, destroying the structure and function of hepatopancreas, causing dose-dependent and time-dependent toxic effects [28]. Andersen et al. found that a high dose of microcystin-LR (MC-LR) could lead to diffuse necrosis and hepatic megalocytosis in the whole liver of *Atlantic salmon* [29]. Previous studies have demonstrated that MCs can be transferred to more sensitive organisms through the food chain/network [30].

NH_4_^+^ can induce the antioxidant defense of juvenile crucian carp. High concentration NH_4_^+^ has toxic effects on CAT, SOD, and glutathione (GSH) in the fish liver [31]. Histopathological changes in the gills, liver, and kidney of *Oreochromis niloticus* are caused by different concentrations of NH_4_^+^, and include gill congestion, telangiectasia, turbid swelling, edema degeneration of liver tissue, kidney congestion, and glomerulonephritis [32]. NH_4_^+^ significantly affects the plasma and hematological parameters of juvenile *Megalabrama amblycephala*, demonstrating histopathological changes in the gills, liver, and kidney of fish. The severity of the lesions is different, with the liver exhibiting the most extensive damage, followed by the gills and kidneys [33].

In addition, it is reported that MCs and NH_4_^+^ have synergistic effects on the immunotoxicity of aquatic organisms. After combined poisoning, the peripheral interspace of the lymphocytes of *Megalabrama amblycephala* is broadened, the nucleus is atrophied, and the mitochondria are swollen. Moreover, the exposure to algae toxin and NH_4_^+^ has a significant interaction with macrophage phagocytosis activity, respiratory burst activities, a total number of white blood cells and the transcriptional levels of *sIgM*, *mIgD,* and *sIgZ* genes of *Megalabrama amblycephala* [23].

### 2.2. Impacts of Cyanobacteria Blooms on Aquatic Flora

The cyanobacteria blooms have strong inhibitory effect on the photosynthetic activity of aquatic flora, leading to leaf death and irreversible inhibition of photosynthesis [34]. Long-term and high-concentration aggregation of cyanobacteria will shade, consume oxygen, and release allelochemicals and MCs, resulting in the disappearance of submerged vegetation [35]. Cyanobacteria blooms lead to the Chla of *Potamongeton malaianus* and *Stuckenia pectinata* decreasing by 50% and 56%, respectively [36].

MCs can induce the reactive oxygen species (ROS) production and an increase in malondialdehyde (MDA), exacerbating the oxidative damage for aquatic flora [37]. MCs can bind irreversibly with phosphatase-1 (PP1) and phosphatase-2A (PP2A) covalently, causing a series of biochemical reactions in cells to be disordered and changing chlorophyll contents and pigment composition in plants [38].

The anatoxin-a produced by cyanobacteria can cause the disorder of oxidative stress reaction in aquatic flora [39]. Treatment with 0.01–0.2 μg/mL MC-LR for 96 h can inhibit the growth of *Spirodela oligorrhiza* [40]. MC-LR concentration of 1.0 μg/L can significantly impede the development of the roots of *Lepidium sativum*, and a concentration of 10 μg/L can inhibit the growth of the whole plant [41]. It has also been found that 0.12–3 μg/mL MCs can hinder the growth of *Oryza sativa* L. [42]. In addition, MCs can cause the gap of aeration tissue in the rhizomes of *Phragmites australis* to be blocked by callus-like tissue, resulting in the gangrene of outer skin tissue in the reed root. When exposed to 10–40 μg/mL of MC-LR for 120 h, the cytoskeleton of reed root changes (microtubule degradation), and its roots swell and deform [43].

MCs can damage DNA and produce genotoxicity. Nuclear shrinkage and chromatin condensation can be observed in the root tip meristem cells of *Phragmites australis* treated with MCs, and chromatin condensation is often accompanied by nuclear shrinkage and apoptosis [44]. DNA damage effect of MCs on *Oryza sativa* root cells by DNA fragmentation and random amplified polymorphic DNA (RAPD) [45]. Furthermore, the affected biochemical processes involved protein folding and stress response, protein biosynthesis, regulation of cell signal and gene expression, and energy and carbohydrate metabolism [46].

The high concentrations of NH_4_^+^ and nitrate nitrogen (NO_3_^−^-N) released by cyanobacteria decay have toxic effects on aquatic plants, resulting in the yellowing of plant leaves, inhibition of growth, and root morphological changes [47]. A high concentration of NH_4_^+^ can also inhibit the absorption of K^+^, Ca^2+^ and Mg^2+^ by plant cells, resulting in a disturbance of ion balance [48]. Studies have also demonstrated that a high concentration of NH_4_^+^ leads to the destruction of the antioxidant system balance of aquatic flora, and the accumulation of ROS, which leads to the damage of plasma membrane [49].

### 2.3. Impact of Cyanobacteria Blooms on Microorganisms in the Aquatic Environment

Studies on the effects of cyanobacteria blooms on microorganisms in water mainly focus on the community structure and activity of microorganisms, especially at the genus level [50]. Cyanobacteria blooms in the summer, and the abundance of *Proteobacteria* in the water and sediment of Zhushan Bay is the highest at the phylum level, followed by Actinomycetes. At the genus level, the dominant bacteria in the water are *GpXI* and *GpIIa*, and the predominant bacteria in the sediment are *Gp6* and *GpIIa* [51]. Meanwhile, the different stages of cyanobacteria blooms will lead to changes in DO, N, and P in surface sediments [52]. Studies have studied and analyzed the bacterial community diversity in Poyang Lake waters and found a specific correlation between DO, Cond, salinity, mineralization, nutrients, and bacterial community diversity index [53]. In addition, debris formed during the degradation of cyanobacteria will precipitate into the surface sediments, stimulating the growth of microorganisms. Studies have demonstrated that the total bacterial diversity of water decreases during cyanobacteria blooms [54]. The decomposition of cyanobacteria will increase the diversity and abundance of ammoniated bacteria in sediments, among which the relative abundance of *Nitrosomonas* *oligotropha* is as high as 75% [55].

Studies have demonstrated that the accumulation of cyanobacteria will lead to a change in microbial community structure and a decrease in diversity in the chironomid larvae gut. The relative abundance of *β-proteobacteria* increased to 40.6%, and the relative abundance of *δ-proteobacteria* decreased to 4.1%. Moreover, cyanobacteria blooms can promote the expression of the *nosZ* gene and increase the abundance of *nirK* denitrifying bacteria [56]. The occurrence of cyanobacteria blooms will lead to the decrease in α-diversity of the bacterial community [57].

## 3. Impacts of Cyanobacteria Blooms on Human Health

Cyanobacteria blooms directly affect drinking water. In 1996, in Caruaru, Brazil, 50 dialysis clinic patients died because of using water contaminated with MCs [58]. In 1999, the cyanobacteria blooms in Dianchi Lake covered an area of 20 km^2^. In May 2007, a massive cyanobacteria bloom in Taihu Lake (Wuxi, China) led to a drinking water crisis for 2 million people in the city of Wuxi [59]. In August 2014, cyanobacteria blooms in Lake Erie increased the concentration of MCs in the drinking water, threatening the drinking water safety of nearly half a million people [60].

When cyanobacteria blooms decompose, releasing many odor substances and cyanotoxins, it has been found that 2-methylisoborneol (MIB) and geosmin are the most common substances that cause odor (musty smell) in drinking water, and their odor threshold concentrations are only 9 and 4 ng/L, respectively [61]. Common substances of odor in water and their relationship with algae products are shown in Table 1. Among the eight kinds of odor in the table, except the chemical taste, chloride taste, and medicinal taste, the other five kinds of odor substances are related to odor compounds produced by algae. Excessive odor content in water affects the quality of drinking water and human health [59].

Cyanobacteria can release toxins such as the hepatotoxin class, neurotoxin, and endotoxin. MCs is the most widely distributed in water, which is a cyclic heptapeptide composed of seven amino acids, mainly produced by *Microcystis* and *Anabaena* [23]. *Microcystis* is the dominant species of cyanobacteria blooms in Taihu Lake, and its biomass can account for 40–98% of the total algae biomass [62]. *Anabaena* is the most common species in cyanobacteria blooms and the only species with hepatotoxic and neurotoxic secondary metabolites [63]. Turner et al. analyzed the MCs of cyanobacteria in freshwater ecosystems in the United Kingdom and found that more than 50% of the water bodies had MCs, and of which about 13% exceeded the World Health Organization (WHO) medium health threshold (20 μg/L) [64].

The WHO has reported that 59% of cyanobacteria causing water blooms in the world are harmful cyanobacteria. More than 80 kinds of MCs have been found [23], among which MC-LR, -RR, and -YR are the most common. The main known algal toxins and hazards are shown in Table 2.

The toxins can be accumulated by organisms and transferred through the food chain/network (Figure 1). Cyanotoxins are chronically toxic to humans, which lead to acute gastroenteritis, respiratory adverse reaction, eye and ear irritation, skin rash, mouth ulcers, and other diseases [10]. In addition, algal toxins can inhibit the synthesis of protein phosphatase, resulting in hyperphosphorylation of critical regulatory proteins in the signal transduction process that controls cytoskeleton tissues [72].

MCs are hydrophilic and soluble in the blood of organisms. They cannot penetrate the lipid membrane through passive diffusion [73]. Therefore, most ingested toxins cannot pass through the ileal epithelium, stay in the digestive tract, and are most likely excreted through feces [74]. However, some studies have demonstrated that ingested MCs can be transported by bile acid membrane transporters (such as organic anion transporters (OATPs)) through the ileum into the venous blood flow and from the portal vein into hepatocytes [75]. The liver is the main target organ for the accumulation and detoxification of MCs. At the same time, MCs can also be detected in other organs (such as the intestine, kidney, brain, lung, and heart), though to a much lesser extent [74]. High doses of cyanobacterial toxins can cause acute liver damage, hepatomegaly, liver hemorrhage, loss of liver cell structure and function, and even biological respiratory arrest [76].

## 4. Conclusions

With the aggravation of global warming and water eutrophication, cyanobacteria blooms are increasing worldwide, and the frequency and duration of cyanobacteria blooms are also increasing. The formation of cyanobacteria blooms has changed the water quality (such as reducing dissolved oxygen and transparency), affected the growth and development of aquatic animals and plants, and changed the community structure and diversity of microorganisms. When people come into direct or indirect contact with water polluted by MCs, it will cause adverse reactions, organ damage, and even death in severe cases. Therefore, it is necessary to propose economical and eco-friendly strategies for the prevention and control of cyanobacteria blooms. Simultaneously, methods for the management of cyanobacteria blooms are constantly being explored.

## Figures and Tables

**Table 1:** Common substances of organoleptic perception in water.

Odor Type		Compounds
Taste odor	Sour	Citric acid, acetic acid
	Sweet	Sucrose, glucose
	Bitter	Caffeine, quinine hydrochloride
	Salty	Sodium chloride
Mouth/nose sensations	Spicy, greasy, spicy Metallic	Aluminium sulfate; menthol; methanol
	Earthy/musty	Geosmin; 2-MIB; IPMP etc.
	Fragrant	4-Nonylphenol; Decanal
	Grassy/woody	cis-3-Hexen-1-ol; cis-3-Hexenylacetic acid; β-Cyclocitric acid
	Fishy	2-trans-4,7-cis-Decatrienal; 2,4-cis-Heptadienal
	Swamp	Dimethyl sulfide compounds; Isopropyl mercaptan
	Chemical	MTBE; 2-EDD etc.
	Chlorinenous	Free chlorine; Monochloramine; Dichloramine
	Medicinal	Bromophenol; Chlorophenols; Iodoform

**Table 2:** Main cyanobacteria toxins.

Toxins	Toxic Effect on Human Health
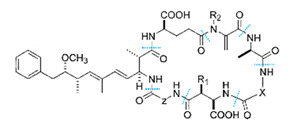 Microcystins	A potent hepatotoxin and tumor promoter, which inhibited protein phosphatase at the molecular level, resulting in hyperphosphorylation of critical regulatory proteins in the signal transduction process of cytoskeletal tissues, resulting in oxidative stress in liver, kidney, brain, and reproductive organs [65].
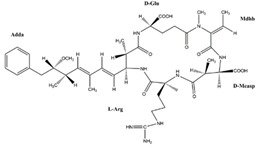 Nodularin	It inhibits the activity of PP-1 and PP-2A, and has tumor-promoting activity, which is considered carcinogenic [66].
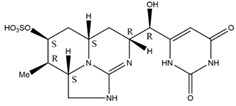 Cylindrospermopsin	Also known as hepatotoxin, it is also harmful to other organs such as the thymus, kidney, and heart [67].
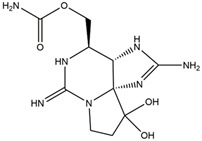 Saxitoxins	A potent neurotoxin, which can be used as an antagonist of voltage-gated sodium channels to motor nerves, causes conduction defects and leads to respiratory paralysis [68].
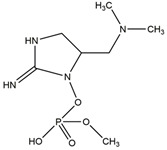 Anatoxin-a	An effective depolarizing neuromuscular blocker, these toxins irreversibly bind to the alkali acetylcholine receptor on the motor nerve endplate, and continuously stimulate muscle cells, thus leading to muscle paralysis [69].
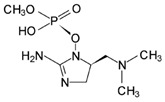 Anatoxin-a(s)	A cholinesterase inhibitor that leads to conduction disturbance and asphyxia death [70]. Anatoxin-a(s) poisoning symptoms include muscle weakness, convulsion, respiratory distress and death due to respiratory failure.
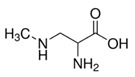 β-N-methylamino-L-alanine, (BMAA)	A developmental neurotoxin, maybe a factor in the increased incidence of Amyotrophic lateral sclerosis (ALS) and Parkinson’s dementia complex (PDC) in Guam [71].

## Data Availability

Not applicable.
